# *N*-oxide alkaloids from *Crinum amabile* (Amaryllidaceae)

**DOI:** 10.3390/molecules23061277

**Published:** 2018-05-26

**Authors:** Luciana R. Tallini, Laura Torras-Claveria, Warley de Souza Borges, Marcel Kaiser, Francesc Viladomat, José Angelo S. Zuanazzi, Jaume Bastida

**Affiliations:** 1 Group of Natural Products, Faculty of Pharmacy, University of Barcelona, Av. Joan XXIII, 27-31, 08028-Barcelona, Spain; lucianatallini@gmail.com (L.R.T.); lauratorras@hotmail.com (L.T.-C.); fviladomat@ub.edu (F.V.); 2 Department of Chemistry, Federal University of Espírito Santo, Av. Fernando Ferrari 514, 29075-915 Vitoria ES, Brazil; warley000@yahoo.com.br; 3 Medicinal Parasitology and Infection Biology, Swiss Tropical Institure, Socinstrasse 57, 4051 Basel, Switzerland; marcel.kaiser@unibas.ch; 4 University of Basel, Petersplatz 1, 4001 Basel, Switzerland; 5 Faculty of Pharmacy, Federal University of Rio Grande do Sul, Av. Ipiranga 2752, 90610-000 Porto Alegre RS, Brazil; zuanazzi@ufrgs.br

**Keywords:** *Crinum amabile*, augustine *N*-oxide, buphanisine *N*-oxide, biological activities

## Abstract

Natural products play an important role in the development of new drugs. In this context, the Amaryllidaceae alkaloids have attracted considerable attention in view of their unique structural features and various biological activities. In this study, twenty-three alkaloids were identified from *Crinum amabile* by GC-MS and two new structures (augustine *N*-oxide and buphanisine *N*-oxide) were structurally elucidated by NMR. Anti-parasitic and cholinesterase (AChE and BuChE) inhibitory activities of six alkaloids isolated from this species, including the two new compounds, are described herein. None of the alkaloids isolated from *C. amabile* gave better results than the reference drugs, so it was possible to conclude that the *N*-oxide group does not increase their therapeutic potential.

## 1. Introduction

Natural products play an important role in the development of new drugs [1]. For example, between 1940 and 2014, 49% of the small molecules approved for the treatment of cancer were developed or directly derived from natural products [1]. The isoquinoline-type alkaloids found in the Amaryllidaceae plant family represent an interesting source of new drugs due to their diverse biological activities [2]. The most important Amaryllidaceae alkaloid is galanthamine, which was approved by the Food and Drug Administration (FDA) for the clinical treatment of mild to moderate Alzheimer’s disease (AD) in 2001, due to its potential acetylcholinesterase inhibitory activity [3]. According to the most recent botanical classification, the Amaryllidaceae are now a subfamily known as the Amaryllidoideae, which together with the Agapanthoideae and Allioideae belong to the Amaryllidaceae family [4]. Amaryllidoideae includes 59 genera and about 850 species, with centers of diversity in South Africa, South America, particularly in the Andean region, and in the Mediterranean [5].

Within the Amaryllidoideae, the pantropical *Crinum* genus is of commercial, economical and medicinal importance [6,7]. This genus contains approximately 65 species, which are widely distributed in diverse habitats, including coastal areas, pans (seasonally flooded depressions), sandy and aquatic areas, and swamps [8]. *Crinum* seeds are highly buoyant, with corky, water-repellent surfaces, allowing them to be dispersed by water [8,9]. Extracts from *Crinum* species have been used in folk medicine to treat fever, pain, swelling, sores, wounds, cancer and malaria [10]. The biological activities of *Crinum* species, including antitumor, immunostimulating, analgesic, antiviral, antibacterial, and antifungal, are attributed to their alkaloid content [7,9].

Known as a decorative plant, *Crinum amabile* has also long been used in Vietnamese folk medicine as an emetic and a remedy for rheumatism and earache [11]. Fifteen alkaloids have been previously identified in *C. amabile*: amabiline, ambelline, augustine, buphanisine, crinamabine, crinamine, crinidine, 4a-dehydroxycrinamabine, flexinine, galanthamine, galanthine, hippeastrine, lycorine, narvedine and tazettine [11,12,13]. Among these, amabiline, augustine, buphanisine, crinamine and lycorine have been isolated from this species and assessed for their antimalarial and cytotoxic potential, with augustine being the most active [13].

Tropical diseases such as malaria, leishmaniasis, Chagas disease and African trypanosomiasis affect more than one billion people and cost developing economies billions of dollars every year [14]. As these diseases prevail in areas where poverty limits access to prevention and treatment interventions, the pharmaceutical industry has little interest in investing in tackling them by drug development [15]. On the other hand, dementia affects around 50 million citizens worldwide, 60–70% of whom suffer from Alzheimer’s disease, for which the current clinical treatment offers only palliative effects [16,17]. Thus, all these diseases require more research on effective treatment, in which the Amaryllidaceae alkaloids may potentially play an important role.

The aim of this work was to perform a detailed study of the alkaloid constituents of *C. amabile*, utilizing spectroscopic and chromatographic methods, including GC-MS and NMR. Two new alkaloids were isolated and chemically characterized by spectroscopic methods and twenty-three known alkaloids were identified by GC-MS. Due to the potential of Amaryllidaceae alkaloids in the clinical treatment of Alzheimer’s disease [3], as well as the activity of augustine against malaria [13], we decided to check the cholinesterase-acetylcholinesterase (AChE) and butyrylcholinesterase (BuChE)-inhibitory activities and the antiprotozoal capacity of six alkaloids isolated from *C. amabile*, including the two new alkaloids. The role of *N*-oxide compounds in these biological activities was explored.

## 2. Results and Discussion

### 2.1. Alkaloids Identified by GC-MS

**Figure 1:**
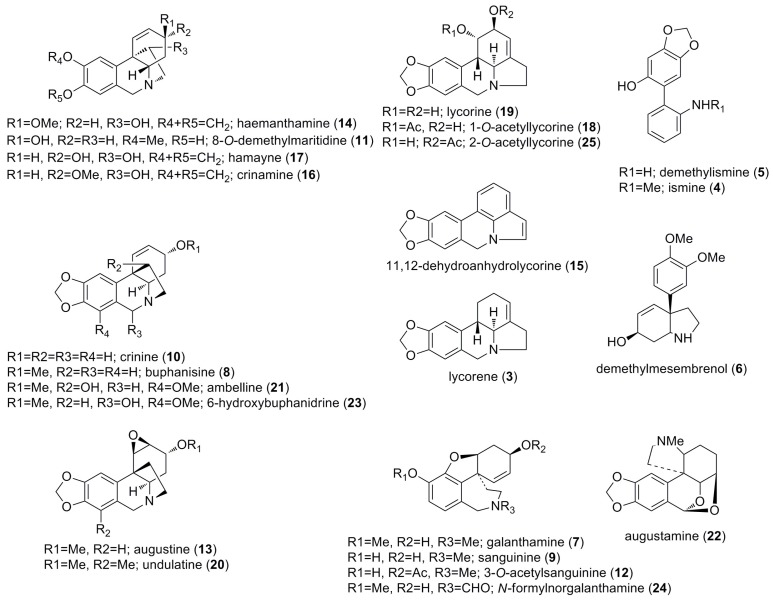
Alkaloids identified in *C. amabile* by GC-MS.

Twenty-three known alkaloids from *Crinum amabile* were identified by GC–MS (Table 1 and Figure 1) by comparison of the Rt, fragmentation patterns and spectral data using our home database. This database was built from single alkaloids isolated and identified by spectroscopic and spectrometric methods (NMR, UV, CD, IR, MS) in the Natural Products Laboratory, University of Barcelona, Spain. Also used were the NIST 05 Database and literature data [18,19,20,21,22].

### 2.2. Structural Elucidation

Two new alkaloids were identified in *C. amabile*: augustine *N*-oxide (**1**) and buphanisine *N*-oxide (**2**), both *N*-oxides of the structures augustine (**13**) and buphanisine (**8**), respectively. *N*-oxides occur as natural products and are not artefacts formed during the isolation procedures [23,24]. Ungiminorine *N*-oxide, homolycorine *N*-oxide, *O*-methyllycorenine *N*-oxide, galanthamine *N*-oxide, sanguinine *N*-oxide, lycoramine *N*-oxide and undulatine *N*-oxide are examples of Amaryllidaceae alkaloid *N*-oxides also reported as natural products [25,26,27,28].

#### 2.2.1. Augustine *N*-oxide (**1**)

The ^1^H-NMR signals of compound **1** (Table 2) were consistent with the structure of augustine [29]. However, H-4α and H-4a and H-6α, H-6β, and H-12*endo* and H-12*exo* were deshielded between 1.21 and 0.36 ppm. These deshielding effects are congruent with the salt or *N*-oxide form of augustine. HR-ESI-MS analysis was carried out to confirm an additional oxygen atom in the structure. Compound **1** exhibited a parent [M + H]^+^ ion at *m/z* 318.1335 in its HR-ESI-MS spectrum, suggesting the molecular formula C_17_H_20_NO_5_ (calcd. 318.1336) and confirming compound **1** as augustine *N*-oxide. 

**Figure 2:**
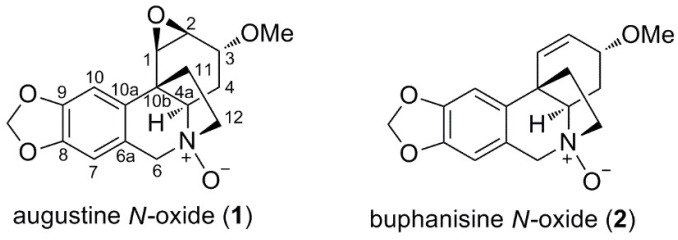
Structures of the two new alkaloids elucidated from *C. amabile*.

The absolute configuration of this structure was determined by CD. The curve and shape were qualitatively similar to those of known crinine-type alkaloids, with the 5,10b-ethano bridge in a β-orientation, having a maximum of around 245 nm and a minimum of 295 nm. The COSY spectrum showed a benzylic coupling between H-7 and H-6, which allowed us to determine the H-7 proton location in the ^1^H-NMR spectrum. The two C-6 protons were differentiated as an AB system with a geminal coupling of around 15.7 Hz. H-4a showed a NOESY correlation with H-6α, which turned out to be crucial for the assignment of its orientation. We were able to determine the H-4β orientation from the large coupling constant between H-4a and H-4β (around 13.8 Hz), and the α-orientation of the methoxy group at C-3 from the small constant between H-4β and H-3 (around 2.7 Hz). The β-orientation of the epoxy group was assignable based on the low values of the H-1 and H-2 constants (3.5 and 3.2 Hz, respectively). The NOESY contour plot between H-4β and H-11*exo* and H-12*exo* allowed us to determine the H-11*exo* and H-12*exo* locations in the ^1^H-NMR spectrum. The quaternary carbons C-6a, C-10a, C-8 and C-9 were ascribed by means of their respective three-bond HMBC correlations with H-10, H-7, H-10 and H-7. Finally, the singlet resonance signal at δ = 43.95 ppm in the ^13^C spectrum was assigned to C-10b, taking into account the three-bond connectivities to H-10, H-4α and H-4β (Figure 2) in the HMBC experiment.

#### 2.2.2. Buphanisine *N*-oxide (**2**)

The ^1^H-NMR spectrum of compound **2** (Table 2) was similar to that of buphanisine [30]. However, the H-4α, H-4a, H-6α, H-6β, H-12*endo* and H-12*exo* protons were assigned as 0.80, 0.24, 0.32, 0.82, 0.86 and 0.29 ppm more deshielded, respectively, than their homologs in buphanisine. HR-ESI-MS analysis allowed us to confirm the presence of *N*-oxide in this structure. Compound **2** exhibited a parent [M + H]^+^ ion at *m/z* 302.1385 in its HR-ESI-MS spectrum, suggesting the molecular formula C_17_H_20_NO_4_ (calcd. 302.1387) and confirming compound **2** as buphanisine *N*-oxide.

The absolute configuration of this structure was determined by CD. The spectrum curve had a maximum of around 245 nm and a minimum of around 292 nm, confirming a crinine-type alkaloid with the 5,10b-ethano bridge in a β-orientation. The assignment of the aromatic protons was based on the benzylic coupling between H-6 and H-7 observed by a 2D COSY experiment. The ^1^H-NMR spectrum showed two doublets at δ 4.84 and 4.72 ppm with a coupling constant of around 15.6 Hz. The first one was assigned as H-6α due to the NOESY contour plot of the proton H-4a. The large coupling constant between H-4a and H-4β (around 13.7 Hz) allowed the H-4β proton location to be determined in the ^1^H-NMR spectrum. The NOESY contour plot between H-4β and H-11*exo* and H-12*exo* enabled us to determine their location in the ^1^H-NMR spectrum. In the HMBC spectrum, the three-bond correlations observed for H-7 to C-9, H-10 to C-8, H-7 to C-10a and H-10 to C-6a allowed us to identify the location of the quaternary carbons C-8, C-9, C-6a and C-10a in the ^13^C spectrum. The three-bond coupling between C-10b and H-2, H-10 and H-4 permitted its location to be assigned at δ = 46.60 ppm in the ^13^C spectrum (Figure 2).

### 2.3. Biological Activities

The alkaloid augustine presents significant activity against chloroquine-sensitive and chloroquine-resistant strains of *Plasmodium falciparum* (IC_50_ = 0.46 and 0.60 µM, respectively) [13]. We consequently decided to verify *in vitro* the activity of six alkaloids isolated from *C. amabile*, augustamine (**22**), augustine (**13**), augustine *N*-oxide (**1**), buphanisine (**8**), buphanisine *N*-oxide (**2**) and crinine (**10**), against four different protozoa, *Trypanosoma brucei rhodesiense*, *Trypanosoma cruzi*, *Leishmania donovani* and *Plasmodium falciparum*, which are related to sleeping sickness, Chagas disease, visceral leishmaniasis and malaria, respectively. These alkaloids are structurally very similar, with the exception of augustamine, which is a unique kind of Amaryllidaceae alkaloid previously isolated from other *Crinum* species, including *C. augustum*, *C. kirkii* and *C. latifolium* [28,31,32], and completely elucidated in 2000 [33]. The rareness of this structure motivated us to isolate it and check its biological activity. In addition, due to the potential effectiveness of Amaryllidaceae alkaloids in the clinical treatment of Alzheimer’s disease, the alkaloids were also tested *in vitro* against acetyl- and butyrylcholinesterase.

#### 2.3.1. AChE and BuChE Inhibitory Activities

All the results for cholinesterase inhibitory activities are shown in Table 3. No tested alkaloid presented BuChE inhibitory activity. AChE inhibitory activity was moderate in augustine (**13**), and low in buphanisine (**8**). The structures of these two alkaloids are very similar: between C-1 and C-2 augustine (**13**) presents an epoxy group and buphanisine (**8**) an olefin group (Figure 1). Interestingly, the augustine (**13**) epoxy group seems to increase the AChE inhibitory activity, more than the olefin in buphanisine (**8**). The AChE inhibitory activity is also slightly improved by the presence of a hydroxyl group at C-3, as occurs in the crinine (**10**) alkaloid, but not by the methoxy group in the same substituent, as occurs in buphanisine (**8**). Unfortunately, the *N*-oxide group did not increase the AChE inhitory activities of augustine *N*-oxide (**1**) and buphanisine *N*-oxide (**2**). Furthermore, augustamine (**22**) did not show any cholinesterase inhibitory activities.

#### 2.3.2. Antiprotozoal Activity

All the alkaloids isolated from *C. amabile* showed low activity against all the protozoa tested (Table 4). Buphanisine (**8**) showed significant inhibitory activity against the NF54 strain of *P. falciparum* (with a 50% inhibitory concentration (IC_50_) of 4.28 ± 0.18 µg mL^−1^. The presence of an *N*-oxide group in augustine *N*-oxide (**1**) and buphanisine *N*-oxide (**2**) appears to decrease their activity against *T. brucei* and *P. falciparum* compared to augustine (**13**) and buphanisine (**8**), respectively. In this experiment, the epoxy group at C-1 and C-2 probably decreases the activity of augustine (**13**) against *P. falciparum* compared to buphanisine (**8**), which has a double bond between C-1 and C-2. Furthermore, the presence of a methoxy group at C-3 seems to increase the activity of buphanisine (**8**) against *P. falciparum* compared to crinine (**10**), which has a hydroxyl group in the same position.

## 3. Materials and Methods

### 3.1. Plant Material

Bulbs of *Crinum amabile* Donn. were collected in Vitoria (Espírito Santo, Brazil) in September 2016. The sample was authenticated by Dr. Alan Meerow at the Subtropical Horticulture Research Station (Miami, FL, USA). A specimen voucher (VIES 39506) has been deposited in the Herbarium of the Universidade Federal do Espirito Santo (UFES; Vitoria, Brazil).

### 3.2. Equipment

About 2 mg of each alkaloid extract was dissolved in 1000 µL of methanol (MeOH) and/or chloroform (CHCl_3_) and injected directly into the GC-MS apparatus (Agilent Technologies, Santa Clara CA, USA) operating in the EI mode at 70 eV. A Sapiens-X5 MS column (30 m × 0.25 mm i.d., film thickness 0.25 µm) was used. The temperature gradient performed was the following: 2 min at 100 °C, 100–180 °C at 15 °C min^−1^, 180–300 °C at 5 °C min^−1^ and 10 min hold at 300 °C. The injector and detector temperatures were 250 °C and 280 °C, respectively, and the flow-rate of carrier gas (He) was 1 mL min^−1^. A split ratio of 1:10 was applied and the injection volume was 1 µL. The alkaloids were identified by GC-MS and the mass spectra were deconvoluted using the software AMDIS 2.64. Kovats retention indices (RI) were recorded with a standard calibration *n*-hydrocarbon mixture (C9–C36) using AMDIS 2.64 software.

^1^H-NMR, ^13^C-NMR, COSY, NOESY, HSQC, and HMBC spectra were recorded on a Bruker 400 MHz Avance III equipped with CryoProbe Prodigy (Bruker, Billerica, MA, USA), using CDCl_3_ as the solvent and tetramethylsilane (TMS) as the internal standard. Chemical shifts are reported in units of δ (ppm) and coupling constants (J) are expressed in Hz. CD, UV and IR spectra were recorded on Jasco-J-810 (Jasco, Easton, MD, USA), Dinko UV2310 (Dinko Instruments, Barcelona, USA) and Thermo Scientific Nicolet iN10 MX spectrophotometers (Thermo Fisher Scientific, Waltham, MA, USA), respectively. HR-ESI-MS spectra were obtained on an LC/MSD-TOF (2006) mass spectrometer (Agilent Technologies) operating in the positive mode, applying 4 kV in the capillary, 175 V in the fragmentor, a gas temperature of 325 °C, and N_2_ as the nebulizing gas (15 psi) and drying gas (flow = 7.0 L min^−1^). Silica gel SDS chromagel 60 A CC (6–35 µm) was used for VLC, and silica gel 60 F_254_ (Merck, Darmstadt, Germany) for analytics and prep. Spots on chromatograms were detected under UV light (254 nm) and by Dragendorff’s reagent stain.

### 3.3. Extraction

Fresh bulbs (2.2 kg) and leaves (1.3 kg) of *C. amabile* were collected and macerated with MeOH (3 × 1.0 L) at room temperature for 4 days. The combined macerate was filtered and the solvent evaporated to dryness under reduced pressure. The bulb and leaf crude extracts (485 and 390 g, respectively) were then acidified to pH 3 with diluted sulfuric acid, H_2_SO_4_ (2%, *v/v*). The neutral material was removed with Et_2_O (3 × 200 mL) and extracted with ethyl acetate (EtOAc) (3 × 200 mL) to obtain the acid EtOAc extracts (4.58 and 2.8 g, respectively). The aqueous solutions were basified up to pH 9–10 with ammonium hydroxide, NH_4_OH (25%, *v/v*) and extracted with *n*-hexane, *n*-Hex (3 × 150 mL) to give the *n*-Hex extracts (1.16 and 0.40 g, respectively, and finally extracted with EtOAc (2 × 200 mL) to obtain the EtOAc extracts (5.11 and 1.15 g, respectively).

The extracts were subjected to a combination of chromatographic techniques, including vacuum liquid chromatography (VLC) [34], Sephadex, thin layer chromatography (TLC) and semi-preparative TLC. The VLC is an effective methodology to rapidly and inexpensively separate large or small quantities of compounds from extracts [35]. A silica gel 60 A (6–35 µm) column was used with a height of 4 cm and a variable diameter according to the amount of sample (2.5 cm for 400–1000 mg; 1.5 cm for 150–400 mg). Alkaloids were eluted with n-Hex containing increasing EtOAc concentrations, followed by neat EtOAc, which was gradually enriched with MeOH (reaching a maximum concentration of 20%, *v/v*). Fractions of 10–15 mL were collected, monitored by TLC (UV 254 nm, Dragendorff’s reagent), and combined according to their profiles. For semi-preparative TLC, silica gel 60F^254^ was used (20 cm × 20 cm × 0.25 mm) together with different solvent mixtures depending on each particular sample (EtOAc:MeOH, 9:1, *v/v*; EtOAc:MeOH, 8:2, *v/v*; or EtOAc:CHCl_3_:MeOH, 6:4:2, *v/v/v*), always in an environment saturated with ammonia. The alkaloids were each identified by GC-MS and the two new alkaloids had their structure elucidated by NMR.

Exclusion chromatography was carried out using a Sephadex LH-20 column (2.5 cm × 40 cm) to clean and separate the alkaloids in the n-Hex bulb extract (1.16 g). It was eluted with 100% MeOH, producing 52 fractions, each one containing about 2 mL, which were monitored by TLC and grouped in four fractions. Fraction 3 (1.00 g) was subjected to a VLC column (2.5 cm × 4.0 cm), starting the elution with 100% n-Hex, and gradually increasing the polarity by adding concentrations of EtOAc up to 100%. The MeOH percentage in the mixture was then increased up to a ratio of EtOAc:MeOH (80:20, *v/v*) and finally, keeping the MeOH percentage stable, the EtOAc percentage was decreased and the CHCl_3_ percentage increased to a ratio of EtOAc:MeOH:CHCl_3_ (60:20:40, *v/v/v*). 48 fractions (100 mL each) were collected, analyzed by TLC and grouped in twelve fractions.

Fraction B (28.5 mg) - eluted with n-Hex:EtOAc (eluted with 70:30 until 60:40, *v/v*), fraction D (26.1 mg)-eluted with n-Hex: EtOAc (40:60, *v/v*), fraction F (40.0 mg) - eluted with EtOAc:MeOH (eluted with 96:4 until 92:8, *v/v*), fraction H (10.5 mg) - eluted with EtOAc:MeOH (eluted with 88:12 until 83:17, *v/v*) and fraction J (25.0 mg) - eluted with EtOAc:MeOH:CHCl_3_ (eluted with 80:20:0 until 71:20:9, *v/v/v*) were subject to different semi-preparative TLC using a mobile phase consisting of EtOAc:MeOH:CHCl_3_ (60:20:40, *v/v/v*) in an environment saturated with ammonia. 12.0 mg of augustamine (22) was isolated from fraction B, 9.1 mg of augustine (13) from fraction D, 6.0 mg of buphanisine (8) from fraction F, 2.9 mg of crinine (10) from fraction H, and 4.0 mg of augustine *N*-oxide (1) and 12.0 mg of buphanisine *N*-oxide (2) from fraction J.

### 3.4. Characterization of Compounds

*Augustine N-oxide* (**1**): Amorphous solid; [α${]}_{D}^{22}$ −24.0 (*c* 0.001, CHCl_3_); UV (MeOH) λmax (log ε): 292.0 (3.69), 240.5 (5.57) nm; CD (MeOH, 20 °C) ∆ε_245_ + 5739, ∆ε_295_ −5169; IR v_max_ 3363, 2986, 2851, 1740, 1504, 1489, 1464, 1391, 1374, 1241, 1147, 1079, 1034, 926, 846 and 814 cm^−1^; ^1^H-NMR (CDCl_3_, 400 MHz) and ^13^C-NMR (CDCl_3_, 100 MHz) see Table 2; ESI-MS data shown in Table 1; HR-ESI-MS of [M + H]^+^ *m/z* 318.1335 (calcd. for C_17_H_20_NO_5_, 318.1336).

*Buphanisine N-oxide* (**2**): Amorphous solid; [α${]}_{D}^{22}$ −1.0 (*c* 0.001, CHCl_3_); UV (MeOH) λmax (log ε): 292.0 (3.57), 240.5 (3.45) nm; CD (MeOH, 20 °C) ∆ε_245_ + 7271, ∆ε_292_ −7223; IR v_max_ 3350, 3037, 2977, 2824, 1652, 1488, 1402, 1377, 1255, 1241, 1097, 1071, 1034, 966, 931 and 854 cm^−1^; ^1^H-NMR (CDCl_3_, 400 MHz) and ^13^C-NMR (CDCl_3_, 100 MHz) see Table 2; ESI-MS data shown in Table 1; HR-ESI-MS of [M + H]^+^ *m/z* 302.1385 (calcd. for C_17_H_20_NO_4_, 302.1387).

### 3.5. Biological Activities

#### 3.5.1. Antiprotozoal Activities

In vitro tests for the biological activity of the alkaloids isolated from *C. amabile* against *Trypanosoma brucei rhodesiense* (trypomastigotes forms, STIB 900 strain), *Trypanosoma cruzi* (axenic grown amastigotes forms, Tulahuen C4 strain), *Leishmania donovani* (amastigotes forms, MHOM-ET-67/L82 strain), and *Plasmodium falciparum* (intraerythrocytic forms, IEF, NF54 strain) and a cytotoxicity test against the mammalian L6 cell line from rat skeletal myoblasts were carried out at the Swiss Tropical and Public Health Institute (Swiss TPH, Basel, Switzerland) according to established protocols as described by Orhan and co-workers [36]. The reference drugs used in these assays were melarsoprol, benznidazole, miltefosine, chloroquine and podophyllotoxin, respectively.

#### 3.5.2. Acetylcholinesterase and Butyrylcholinesterase Inhibitory Activities

Cholinesterase inhibitory activities were analyzed as by Ellman and co-workers [37] with some modifications as by López and co-workers [38]. Fifty microliters of AChE or BuChE phosphate buffer (8m M K_2_HPO_4_, 2.3 mM NaH_2_PO_4_, 0.15 M NaCl, pH 7.5) and 50 μL of the sample dissolved in the same buffer were added to the wells. The plates were incubated for 30 min at room temperature before 100 μL of the substrate solution (0.1 M Na_2_HPO_4_, 0.5 M DTNB, and 0.6 mM acetylthiocholine iodide, ATCI, or 0.24 mM butyrylthiocholine iodide, BTCI, in Millipore water, pH 7.5) was added. The absorbance was read in a Labsystem microplate reader (Helsinki, Finland) at 405 nm after 10 min. Galanthamine served as positive control. In a first step, samples were assessed at 10, 100 and 200 µg mL^−1^ towards both enzymes. Samples with an IC_50_ > 200 µg mL^−1^ were considered inactive. Samples with an IC_50_ < 200 µg mL^−1^ were further analyzed to determine the IC_50_ values. Enzyme activity was calculated as a percentage compared to an assay using a buffer without any inhibitor. The cholinesterase inhibitory data were analyzed with the software Microsoft Office Excel 2010.

## 4. Conclusions

Twenty-five alkaloids were identified in *C. amabile*, including two new alkaloids, augustine *N*-oxide and buphanisine *N*-oxide. This is the first time that augustamine and *N*-oxide structures have been described in this species. These alkaloids, together with augustine, buphanisine and crinine, were isolated, but none showed remarkable biological activity.

## Figures and Tables

**Table 1:** Alkaloids identified in *Crinum amabile* by GC-MS.

Alkaloid	RI	M^+^	MS
Lycorene (**3**)	2102.2	255 (52)	254 (100), 227 (17), 226(20), 211 (15), 183(14), 181(10)
Ismine (**4**)	2124.3	257 (28)	239(16), 238 (100), 196 (10), 168 (10)
Demethylismine (**5**)	2128.8	243 (22)	225(21), 224 (100), 167 (10), 166 (15), 154 (11), 77 (12)
Demethylmesembrenol (**6**)	2177.0	275 (5)	206 (9), 205 (76), 115 (6), 70 (100)
Galanthamine (**7**)	2262.8	287 (85)	286 (100), 244(29), 216 (45), 174(39), 165(16), 141 (14), 128 (21), 115 (31)
Buphanisine (**8**)	2283.7	285 (95)	273 (54), 272 (43), 254 (40), 215 (100), 157 (39), 129 (35), 128 (55), 115 (64)
Sanguinine (**9**)	2288.3	273 (100)	272 (85), 202 (40), 165(20), 160 (50), 131 (20), 128 (19),115 (28), 77(20),
Crinine (10)	2326.7	271 (100)	228 (24), 200 (30), 199(81), 187 (76), 173 (28), 129 (34), 128(44), 115 (47), 56 (32)
8-*O*-Demethylmaritidine (**11**)	2373.8	273 (100)	230 (25), 202 (29), 201 (80), 189 (65), 175 (29), 129 (24), 128 (30), 115 (32), 56 (30)
3-*O*-Acetylsanguinine (**12**)	2387.1	315 (37)	256 (100), 255 (42), 254 (37), 212(26), 165 (33), 152 (23), 115 (30), 96 (67)
Augustine (**13**)	2411.6	301 (93)	228 (36), 187 (30), 175 (300), 173 (24), 159 (38), 143 (57), 128 (259, 115 (75)
Buphanisine *N*-oxide (**2**)	2429.8	301 (nv)	285 (100), 270 (33), 254 (35), 216 (21), 215 (82), 201 (24), 157 (20), 128 (22)
Haemanthamine (**14**)	2436.9	301 (55)	257 (54), 227 (80), 225 (98), 224(80), 181 (100), 153 (46), 152 (46), 115 (64)
11,12-Dehydroanhydrolycorine (**15**)	2448.5	249 (55)	248 (100), 191 (13), 190 (31), 189 (11), 95 (14)
Crinamine (**16**)	2497.6	273 (17)	272 (100), 242 (12), 214 (11), 186 (12), 128 (15)
Hamayne (**17**)	2551.7	259 (14)	258 (100), 242 (11), 214 (10), 211 (12), 181 (14), 128 (19)
1-*O*-Acetyllycorine (**18**)	2563.1	329 (20)	299(15), 268 (28), 250 (17), 244 (20), 227 (56), 226 (100), 240 (11)
Augustine *N*-oxide (**1**)	2571.8	317 (nv)	301 (100), 228 (34), 187 (22), 175 (77), 173 (17), 159 (27), 143 (37), 115 (37)
Lycorine (**19**)	2592.2	287 (19)	286 (13), 268 (18), 250 (10), 227 (60), 226 (100), 147 (11)
Undulatine (**20**)	2594.4	331 (100)	258 (37), 219 (22), 217 (36), 205 (71), 203 (37), 189 (43), 173 (39), 115 (35)
Ambelline (**21**)	2621.1	331 (69)	287 (100), 260 (81), 257 (62), 255 (74), 254 (52), 241 (51), 239 (61), 211 (69)
Augustamine (**22**)	2628.7	301 (76)	300 (100), 245(16), 244(84), 215(33), 201 (32), 188 (14), 115 (22), 70 (21)
6-Hydroxybuphanidrine (**23**)	2631.3	331 (35)	277 (16), 276 (100), 261 (30), 218 (17), 217 (23), 216 (24), 115 (18), 56 (25)
*N*-Formylnorgalanthamine (**24**)	2649.1	301 (100)	225 (26), 211 (29), 181 (19), 165 (14), 129 (18), 128 (22), 115 (30), 77 (15)
2-*O*-Acetyllycorine (**25**)	2676.2	329 (21)	328 (17), 270 (40), 269 (72), 268 (100), 252 (43), 250 (73), 227 (27), 226 (67)

* not visible.

**Table 2:** NMR data for compounds **1** and **2** (400 MHz for ^1^H and 100 Hz for ^13^C, CDCl_3_).

		1		2
No.	δ_C_, type	δ_H_ (*J* in Hz)	δ_C_, type	δ_H_ (*J* in Hz)
1	52.06	3.68, d (3.5)	129.59	6.39, d (10.0)
2	55.06	3.42, ddd (3.2, 2.4, 0.7)	127.18	6.08, ddd (10.0, 5.3, 1.0)
3	73.43	4.12, dd (2.7, 2.5)	71.38	3.95, ddd (5.6, 3.6, 2.0)
4α	19.72	2.91, dt (14.1, 3.1)	23.46	3.13 ddt (13.6, 4.2, 2.4)
4β	19.72	1.54, ddd (13.8, 13.8, 2.9)	23.46	1.72 ddd (13.7, 13.7, 4.0)
4a	72.59	3.51, dd (13.4, 3.6)	74.14	3.74, dd (13.2, 4.3)
6α	67.56	4.83, dd (15.7, 1.8)	76.55	4.84, d (15.6)
6β	67.56	4.68, d (15.7)	76.55	4.72, d (15.6)
6a	122.33	-	121.71	-
7	106.40	6.57, s	106.44	6.54, s
8	147.13	-	147.20	-
9	147.79	-	147.79	-
10	102.49	6.90, s	102.98	6.81, s
10a	133.92	-	134.75	-
10b	43.95	-	46.60	-
11endo	35.64	1.99, ddd (12.4, 9.4, 5.1)	40.02	2.11, ddd (12.5, 8.0, 6.0)
11exo	35.64	2.79, ddd (12.4, 12.4, 6.9)	40.02	2.26, ddd (12.2, 10.8, 8.6)
12endo	67.56	3.81, ddd (12.8, 9.4, 7.0)	68.97	3.88, m
12exo	67.56	3.73, dddd (12.5,12.5, 5.0, 2.2)	68.97	3.85, m
OCH_2_O	101.58	5.99, d (1.3), 5.98 d (1.3)	101.51	5.95, d (1.3), 5.93 d (1.3)
OMe	57.92	3.47, s	57.23	3.39, s

**Table 3:** Results of AChE and BuChE inhibitory activities of the alkaloids isolated from *C. amabile*.

Alkaloid	AchE ^*^	BuChE ^*^
Augustine *N*-oxide (**1**)	79.64 ± 5.26	>200
Buphanisine *N*-oxide (**2**)	>200	>200
Agustamine (**22**)	>200	>200
Augustine (**13**)	45.26 ± 2.11	>200
Buphanisine (**8**)	183.31 ± 36.64	>200
Crinine (**10**)	163.89 ± 15.69	>200
Galanthamine (**7**)	0.45 ± 0.03	3.88 ± 0.19

^*^ all results are in µg mL^−1^.

**Table 4:** *In vitro* antiprotozoal and cytotoxic activities of **1** and **2**. Values expressed in IC_50_ (µg mL^−1^).

Parasite	*T. brucei rhodesiense*	*T. cruzi*	*L. donovani*	*P. falciparum*	Cytotoxicity
Reference drug	0.003 ± 0.001 ^a^	0.865 ± 0.08 ^b^	0.515 ± 0.06 ^c^	0.004 ± 0.0007 ^d^	0.004 ± 0.0007 ^e^
Augustine (**13**)	15.05 ± 1.06	56.00 ± 0.71	>100	14.20 ± 0.14	>100
Augustine *N*-oxide (**1**)	58.85 ± 11.53	66.25 ± 11.81	>100	36.65 ± 4.74	>100
Buphanisine (**8**)	16.5 ± 0.57	55.55 ± 4.60	>100	4.28 ± 0.18	72.85 ± 5.02
Buphanisine *N*-oxide (**2**)	55.25 ± 4.31	64.05 ± 1.34	>100	32.55 ± 0.07	>100
Crinine (**10**)	18.95 ± 0.78	57.45 ± 6.86	>100	30.95 ± 2.19	>100
Augustamine (**22**)	19.20 ± 2.97	54.00 ± 4.53	>100	20.35 ± 0.21	81.55 ± 0.64

^a^ melarsoprol; ^b^ benznidazole; ^c^ miltefosine; ^d^ chloroquine; ^e^ podophyllotoxin.
